# Deep Plant Phenomics: A Deep Learning Platform for Complex Plant Phenotyping Tasks

**DOI:** 10.3389/fpls.2017.01190

**Published:** 2017-07-07

**Authors:** Jordan R. Ubbens, Ian Stavness

**Affiliations:** Department of Computer Science, University of SaskatchewanSaskatoon, SK, Canada

**Keywords:** phenotyping, deep learning, methods, computer vision, machine learning

## Abstract

Plant phenomics has received increasing interest in recent years in an attempt to bridge the genotype-to-phenotype knowledge gap. There is a need for expanded high-throughput phenotyping capabilities to keep up with an increasing amount of data from high-dimensional imaging sensors and the desire to measure more complex phenotypic traits (Knecht et al., 2016). In this paper, we introduce an open-source deep learning tool called Deep Plant Phenomics. This tool provides pre-trained neural networks for several common plant phenotyping tasks, as well as an easy platform that can be used by plant scientists to train models for their own phenotyping applications. We report performance results on three plant phenotyping benchmarks from the literature, including state of the art performance on leaf counting, as well as the first published results for the mutant classification and age regression tasks for *Arabidopsis thaliana*.

## 1. Introduction

The genotype-to-phenotype gap is one of the most important problems in modern plant breeding (Houle et al., 2010; Großkinsky et al., 2015). While genomics research has yielded much information about the genetic structure of various plant species, sequencing techniques and the data they generate far outstrip our current capacity for plant phenotyping (Yang et al., 2014). Traditional plant phenotyping tools, which rely on manual measurement of selected traits from a small sample of plants, have very limited throughput and therefore prevent comprehensive analysis of traits within a single plant and across cultivars. This so-called *phenotyping bottleneck* (Furbank and Tester, 2011) limits our ability to understand how expressed phenotypes correlate with underlying genetic factors and environmental conditions and has slowed progress in important breeding problems such as drought resistance (Großkinsky et al., 2015).

Image-based techniques have potential to vastly increase the scale and throughput of plant phenotyping activities. Through a combination of new imaging technologies, robotic and conveyer-belt systems in greenhouses, and ground-based and aerial imaging platforms in fields, the capacity to take pictures of plants and crops has expanded dramatically in the past 5 years (Fahlgren et al., 2015b). However, a key requirement for image-based phenotyping tools is to automatically transform those pictures into reliable and accurate phenotypic measurements. In addition, these tools must be capable of measuring a wide variety of phenotypes to allow for flexibility and relevance to a range of scientific applications.

It has been proposed that future progress in image-based plant phenotyping will require a combined effort in the domains of image processing for feature extraction and machine learning for data analysis (Tsaftaris et al., 2016). In the current machine learning literature, deep learning methods lead the state of the art in many image-based tasks such as object detection and localization, semantic segmentation, image classification, and others (LeCun et al., 2015). Deep learning methods in computer vision, such as deep convolutional neural networks, integrate image feature extraction with regression or classification in a single pipeline which is trained from end to end simultaneously (LeCun et al., 1990). However, few deep learning applications have been demonstrated in the plant phenotyping literature, and no general purpose tools have been presented to the plant phenotyping community to support and promote these methods.

In this paper, we present an open-source software platform, Deep Plant Phenomics, which implements deep convolutional neural networks for the purpose of plant phenotyping. We demonstrate the effectiveness of our approach in three complex phenotyping tasks described in the literature: leaf counting, mutant classification, and age regression for top-down images of plant rosettes. Our goal is to provide the plant phenotyping community access to state-of-the-art deep learning techniques in computer vision in order to accelerate research in plant phenotyping and help to close the genotype-to-phenotype gap.

## 2. Background

### 2.1. Image analysis for plant phenotyping

**Figure 1:**
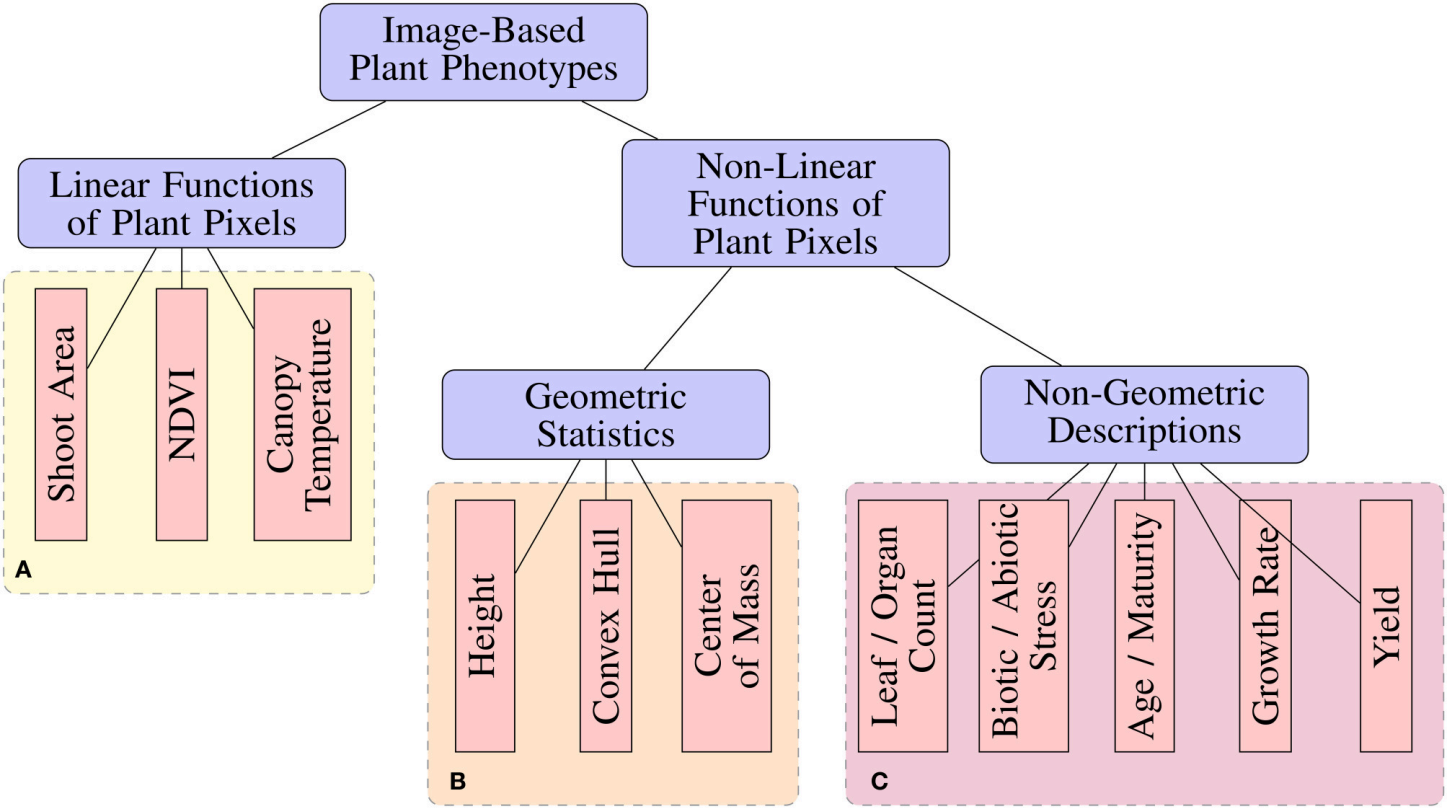
A taxonomy of image-based plant phenotyping tasks. Phenotypes in **(A,B)** can be accurately measured using classical image processing techniques (Fahlgren et al., 2015b), while those in **(C)** are complex phenotyping tasks requiring more sophisticated analyses.

Many image analysis tools have been released by the scientific community for the purpose of performing image-based high-throughput plant phenotyping (Hartmann et al., 2011; Fahlgren et al., 2015a; Rahaman et al., 2015; Knecht et al., 2016). These tools range in the degree of automation, as well as the types of phenotypic features or statistics they are capable of measuring. From an image analysis perspective, phenotypic features can be categorized based on their complexity (Figure 1). Image-based phenotypes can be broadly separated into those that are simply linear functions of image pixel intensities or more complex types that are non-linear functions of pixel intensities, which can be either geometric or non-geometric descriptions.

Standard image processing pipelines have provided acceptable results for measuring *Linear* phenotypic features (Figure 1) under controlled experimental conditions. For example, biomass can be estimated from shoot area by segmenting a plant from a known background (Leister et al., 1999). Likewise, accurate measurement of Normalized Difference Vegetation Index (NDVI) (Walter et al., 2015), chlorophyll responses (Campbell et al., 2015), and other simple features have been demonstrated. *Non-linear, geometric* phenotypic features, such as compactness and diameter of rosettes (De Vylder et al., 2012), can be measured as a straight-forward processing step after plant segmentation. However, simple image processing pipelines tend to breakdown when faced with more complex *non-linear, non-geometric* phenotyping tasks. Tasks such as leaf/pod/fruit counting, vigor ratings, injury ratings, disease detection, age estimation, and mutant classification add a higher level of abstraction which requires a more complicated image processing pipeline with several more steps such as morphological operations, connected components analysis, and others (Pape and Klukas, 2015). Not only is this process dataset-specific and labor-intensive, the added complexity contributes additional parameters and potential fragility to the pipeline.

In addition to being limited to simple features, existing image-based phenotyping tools are often also only applicable for processing pictures of individual plants taken under highly controlled conditions, in terms of lighting, background, plant pose, etc. Most tools rely on hand-engineered image processing pipelines, typically requiring the hand-tuning of various parameters. In some circumstances, hand-tuned parameters can be invalidated by variation in the scene including issues like lighting, contrast, and exposure (Li et al., 2014). As such, moving existing image analysis tools out of the laboratory and into the field, where lighting, background, plant overlap, and plant motion cannot be controlled, may prove difficult.

Machine learning techniques, and deep learning in particular, have potential to improve the robustness of image-based phenotyping and extend toward more complex and abstract phenotypic features. By creating high-throughput systems which reach beyond basic phenotypic features, researchers will be able to explore more complex phenotypes which may be useful for genotype-phenotype association. For example, it has been shown in the literature that a collection of automatically measured phenotypes such as tiller count and plant compactness yielded more trait loci in *O. satvia* than did manual measurements of shoot weight and leaf area (Yang et al., 2014).

### 2.2. Deep learning

In response to the limited flexibility and poor performance of classical image processing pipelines for complex phenotyping tasks, machine learning techniques are expected to take a prominent role in the future of image-based phenotyping (Tsaftaris et al., 2016). Plant disease detection and diagnosis is an example of a complex phenotyping task where machine learning techniques, such as support vector machines, clustering algorithms, and neural networks, have demonstrated success (Singh et al., 2016).

Deep learning is an emerging area of machine learning for tackling large data analytics problems. Deep convolutional neural networks (CNNs) are a class of deep learning methods which are particularly well-suited to computer vision problems. In contrast to classical approaches in computer vision, which first measure statistical properties of the image as features to use for learning a model of the data, CNNs actively learn a variety of filter parameters during training of the model. CNNs also typically use raw images directly as input without any time-consuming, hand-tuned pre-processing steps. CNNs and their variants have been shown to substantially out-perform classical machine learning approaches for tasks such as handwriting recognition (LeCun et al., 1990), image classification (He et al., 2016), and instance detection and segmentation (Girshick, 2015).

Given the success in other areas, deep learning has been proposed as a future trend in image-based plant phenotyping (Tsaftaris et al., 2016). Early results from the few studies that have applied the technique are promising: CNNs were effective for plant disease detection and diagnosis (Mohanty et al., 2016) and for classifying fruits and flowers of plants in field images (Pawara et al., 2017). The performance of deep learning in these contexts motivates the present work investigating deep learning for other complex phenotyping tasks, such as leaf counting and morphological classification.

### 2.3. Convolutional neural networks

**Figure 2:**
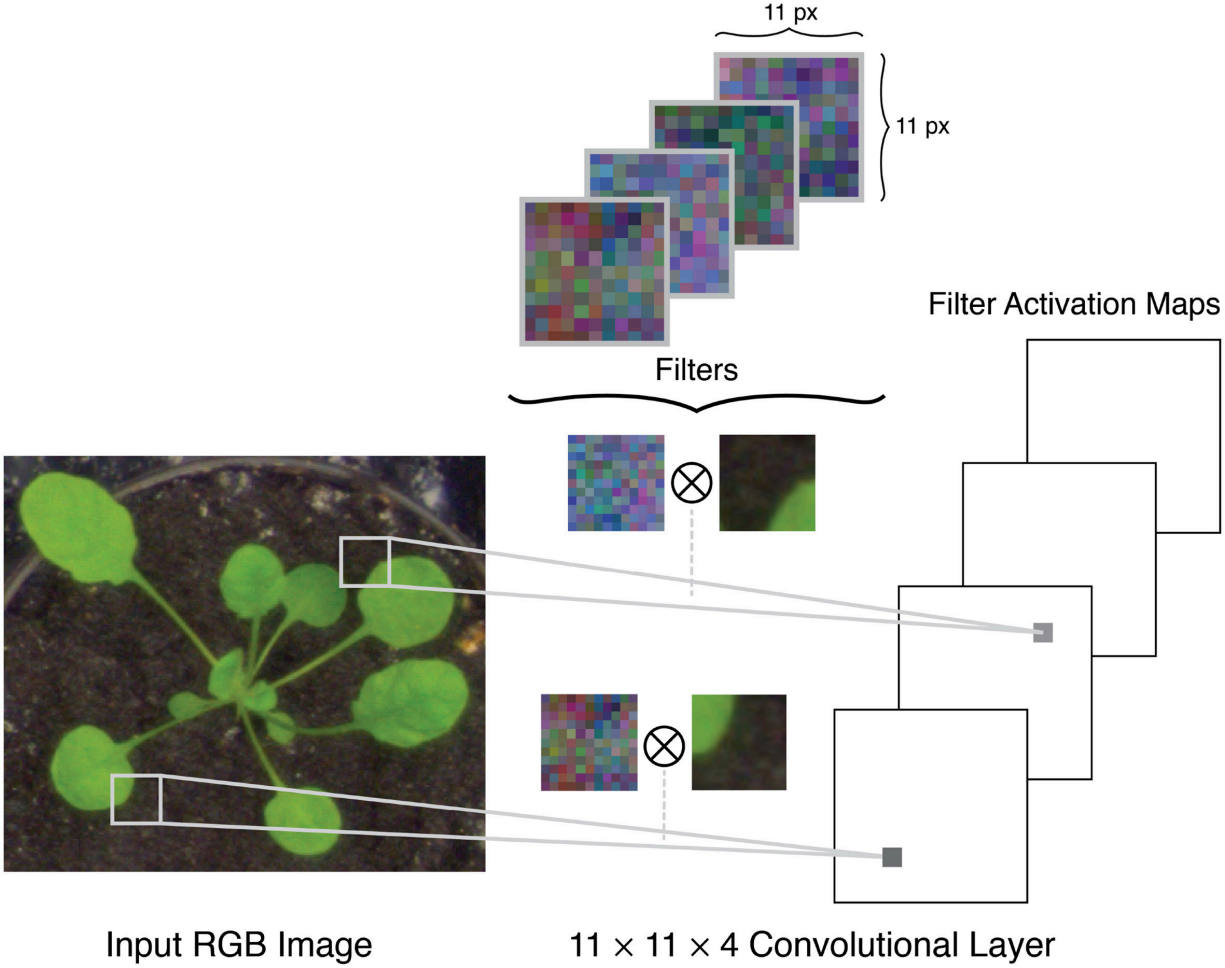
Example of filters in the first convolutional layer of a network being applied to the input image in stride convolutions.

A typical setup for a CNN uses a raw RGB image as input, which can be considered as an *n*×*m*×3 volume, where *n* is the image height, *m* is the image width, and 3 is the number of color channels in the image, e.g., red, green, and blue channels. The architecture of a CNN is comprised of several different layers of three main types: convolutional layers, pooling layers, and fully connected layers. The initial layers in a network are convolutional and pooling layers. The convolutional layers apply a series of filters to the input volume in strided convolutions (Figure 2). Each filter is applied over the full depth of the input volume, and each depth slice in the layer's output volume corresponds to the activation map of one of these filters. For example, if padding is applied at the boundaries of the image and with a stride size of one pixel, the output of the convolutional layer will be an *n*×*m*×*k* volume where *n* and *m* are the height and width of the input volume, and *k* is the number of filters. The pooling layers apply a spatial downsampling operation to the input volume, by calculating the maximum (called *max pooling*) or mean (*average pooling*) value in a pixel's neighborhood.

After a series of convolutional and pooling layers, there are typically one or more fully connected layers, including the output layer. The input to the first fully connected layer is the output volume from the previous layer, which is reshaped into a large one-dimensional feature vector. This feature vector is matrix-multiplied with the weights of the fully connected layer, which produces *pre-activations*. After each convolutional and fully connected layer (with the exception of the output layer), a non-linear function (such as a sigmoid function or *Rectified Linear Unit*) is applied to arrive at the final activations for the layer. The output layer is a fully connected layer without an activation function. In the case of classification, the number of units in the output layer corresponds to the number of classes. These values can then be log-normalized to obtain class probabilities for classification problems. In the case of regression problems, the number of units in the output layer corresponds to the number of regression outputs (for example, one output for leaf count, or four outputs for bounding boxes).

As with other supervised methods, CNNs are trained via an iterative optimization procedure to minimize the difference between the network's output and a known ground-truth label for each input. A loss function (such as *cross-entropy loss*) compares the output value (or values) of the network to the ground-truth label. This results in a singular loss value, which is then back-propagated through the network in reverse order. At each layer, the gradient of the error signal with respect to each parameter (in the weight matrix of the fully connected layers, or the filter weights of the convolutional layers) can be calculated. These parameters can then be adjusted by a factor proportional to this gradient.

## 3. Methods

### 3.1. Software and algorithm

We have created the Deep Plant Phenomics (DPP) platform as an open-source, freely available tool for the plant phenotyping community with the hope to accelerate research results in the area of deep learning for advanced phenotyping. This platform provides an accessible programming interface using the Python language for training models to perform regression and classification tasks, as well as offering pre-trained networks for different plant phenotyping tasks. Deep Plant Phenomics is available for download at https://github.com/usaskdapper/deepplantphenomics. Detailed documentation describing installation and usage of the platform is available in the software repository. For the benchmark tasks discussed in the present paper, we implement deep convolutional neural networks using DPP.

DPP integrates Google's open-source Tensorflow computational library (Abadi et al., 2015). This allows the platform to run on a variety of hardware, including CPUs and GPUs, as well as CPU and GPU clusters. This seamless extensibility from entry-level desktop computers to large compute clusters is important for high-throughput phenotyping, since throughput can be scaled to meet demand (Klukas et al., 2014). The open-source PlantCV library (Fahlgren et al., 2015a) is used in the platform to provide image processing capabilities. The PlantCV module, in conjunction with a pre-trained bounding box regression network, provides automatic segmentation of images from Lemnatec plant scanners, as a demonstration of the potential image processing applications of the package. Multiple dataset loading functions are provided in the platform, including loaders for bounding box coordinates supplied in Pascal VOC format (Everingham et al., 2010), regression and classification labels, CSV files, directories, as well as loaders for the International Plant Phenotyping Network (IPPN) phenotyping dataset (Minervini et al., 2014) and other plant phenotyping datasets.

DPP includes pre-trained neural networks for the rosette leaf counting task as well as the Arabidopsis mutant classification task discussed here. These models can be applied to images with a single line of code. In addition, new models trained using DPP can easily be packaged for deployment in the same manner.

When training new models using the platform, there exists support for DropOut layers (Srivastava et al., 2014), local response normalization layers (Krizhevsky et al., 2012), data augmentation options, data fusion for integrating image meta-data, different optimization and weight initialization schemes, multithreading, regularization, and other tools. These features make the package a powerful and flexible learning platform which can be suited to many phenotyping tasks.

### 3.2. Dataset and tests

For the three experiments presented in this paper, the IPPN image-based plant phenotyping dataset was used (Minervini et al., 2015). This dataset includes multiple computer vision benchmarks for tasks such as plant and leaf segmentation, leaf counting, classification, and others. This image dataset has been extensively studied as it has been the subject of competitions in leaf segmentation and leaf counting.

**Figure 3:**
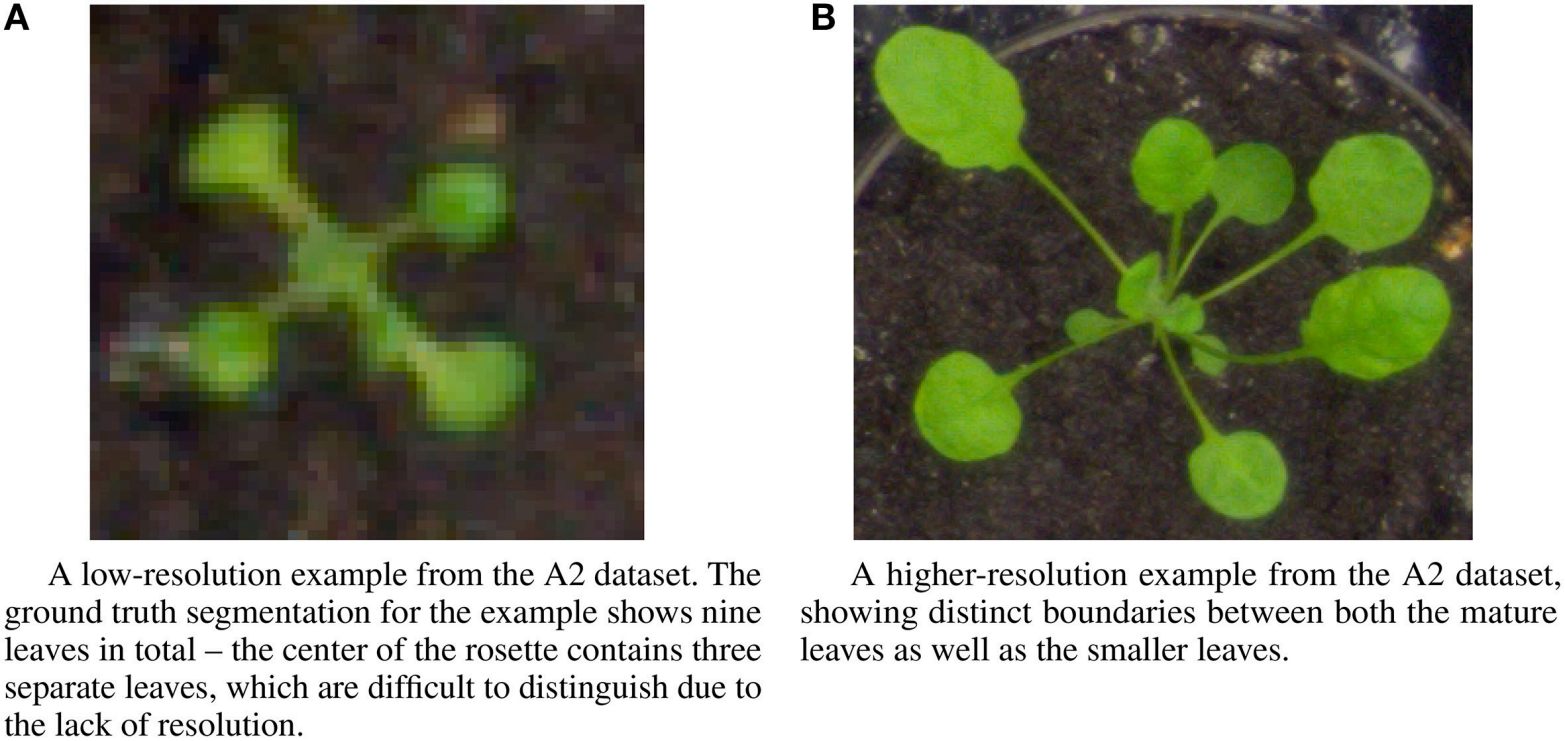
Example images from the A2 dataset showing a range of diversity in image resolution, leaf size and shape, and leaf counts. **(A)** A low-resolution example from the A2 dataset. The ground truth segmentation for the example shows nine leaves in total—the center of the rosette contains three separate leaves, which are difficult to distinguish due to the lack of resolution. **(B)** A higher-resolution example from the A2 dataset, showing distinct boundaries between both the mature leaves as well as the smaller leaves.

The IPPN dataset includes several different image sets multiple contexts: images of individual plants, and trays containing multiple plants. For the experiments described here, we focus on the images of individual plants, which are subdivided into three datasets—two datasets of *Arabidopsis thaliana* (A1, A2) and one dataset of *Nicotiana tabacum* (tobacco) (A3). We perform the leaf counting task on all three datasets, as well as the mutant classification and age regression tasks on the A2 Arabidopsis dataset for which ground truth is available. All tasks use only the RGB images from each of the three datasets. The sizes of each of the three datasets are 120, 165, and 62 examples, respectively. Examples from the A2 dataset show a wide variation in image resolution, the size of the visual field, as well as morphological differences such as leaf shape and size (Figure 3). The number of leaves varies between five and twenty leaves per plant for the Arabidopsis examples, and between two and thirteen for the Tobacco examples. To determine the ground truth for leaf counts, the authors of the dataset extrapolated the count from human expert provided leaf segmentations for each image. Further description of the dataset and the methodology used in its construction is provided in the publication (Minervini et al., 2015).

The phenotyping tasks evaluated in the present study represent challenging traits to measure from images. Leaf count is an important phenotype because of its correlation with such features as yield, drought tolerance, and flowering time (Minervini et al., 2015). This makes leaf count not only distinct from shoot area or biomass, but a useful phenotype in its own right. Mutant classification is related to identifying morphological differences between plant varieties. While an experiment may not explicitly want to classify mutants (as these would already be known), classifying images of plants based on morphological differences is important because the same morphological changes to a plant that are observed (induced) in a mutant may be relevant phenotypes for natural plants, i.e., the morphological changes present in certain mutants may be caused by other pathways such as from pests or disease; therefore mutant classification can be a demonstration of more challenging disease classification that have morphological features rather than color, etc. Age regression, measured in hours after germination, relates to plant maturity, which is an important phenotype in plant breeding. While an experiment may not directly need to estimate age, since it is known *a* priori, estimating the maturity of different varieties is important. For example, which variety matures earlier or more rapidly at certain growth phases.

In order to demonstrate that the proposed method is robust to changes in scene lighting, an additional experiment was performed on the A2 leaf counting dataset. In this robustness experiment, the brightness and contrast of images in the test were randomly adjusted. Since the model is also trained with brightness and contrast adjustments as a form of augmentation (detailed below), different parameters for this adjustment were used to bring the distortions out of the range seen by the model during training. During the training, brightness was modified with a maximum delta of 63 and contrast was modified with a lower range of 0.2 and an upper range of 1.8. For testing, the delta for brightness was set to 75, and the lower and upper parameters for contrast were set to 0.5 and 2.1, respectively.

### 3.3. Approach

Convolutional neural networks (CNNs) were constructed and trained from scratch to perform each of the three benchmark tasks. The structure of the network varied slightly between tasks, as the model was tailored to the problem and the data (Table 1). This tailoring of the architecture is not necessary; however, we perform the modifications here in order to obtain higher performance results and demonstrate the capabilities of the method.

**Table 1:** The network architectures used for each of the phenotyping datasets and tasks.

	**A1**	**A2**	**A3**	**Mutant**	**Age**
Input size	256 × 256	128 × 128	256 × 256	128 × 128	128 × 128
	Conv 5 × 5	Conv 5 × 5	Conv 5 × 5	Conv 5 × 5	Conv 3 × 3
	Pool 3 × 3	Pool 3 × 3	Pool 3 × 3	Pool 3 × 3	Pool 3 × 3
	Conv 5 × 5	Conv 5 × 5	Conv 5 × 5	Conv 5 × 5	Conv 3 × 3
	Pool 3 × 3	Pool 3 × 3	Pool 3 × 3	Pool 3 × 3	Pool 3 × 3
	Conv 3 × 3	Conv 3 × 3	Conv 3 × 3	Conv 5 × 5	FC 2048
	Pool 3 × 3	Pool 3 × 3	Pool 3 × 3	Pool 3 × 3	Output 1
	Conv 3 × 3	Conv 3 × 3	Conv 3 × 3	Conv 5 × 5	
	Pool 3 × 3	Pool 3 × 3	Pool 3 × 3	Pool 3 × 3	
	Conv 3 × 3	Conv 3 × 3	Conv 3 × 3	FC 4096	
	Pool 3 × 3	Pool 3 × 3	Pool 3 × 3	DropOut (0.5)	
	Conv 3 × 3	Output 1	Conv 3 × 3	FC 4096	
	Pool 3 × 3		Pool 3 × 3	DropOut (0.5)	
	FC 1024		Output 1	FC 4096	
	Output 1			Output 5	
				Softmax	

*All pooling operations are max pooling with a stride of 2*.

For the A2 leaf counting dataset, a convolutional neural network was constructed with two 5 × 5 convolutional layers, three 3 × 3 convolutional layers, and an output layer. Each convolutional layer was followed by a max pooling layer with a 3 × 3 spatial size and a stride of 2 pixels. The Xavier (Glorot) initialization scheme (Glorot and Bengio, 2010) was used in each case, with *tanh* used as the activation function. Images were resized to 128 × 128 and cropped to 96 × 96 randomly during training, and to center during testing. For all experiments, the only pre-processing applied to the images was per-image standardization, which subtracts the mean from the image matrix and divides by the standard deviation.

The A1 dataset includes only one accession of Arabidopsis (Col-0), which tends to have smaller and more tightly packed leaves. Therefore, we increased the input size to 256 × 256 pixels and added an additional 3 × 3 convolutional and pooling layer to the network. We reduced the automatic cropping from 25 to 10% to avoid losing leaves near the edges of the image, as images in this dataset seem to be more tightly cropped. We also added a fully connected layer with 1,024 units. For the A3 dataset, we used the same modifications as for the A1 dataset, with the exception of the fully connected layer.

For the mutant classification task, the network used a feature extractor comprised of four 5 × 5 convolutional layers, each followed by a pooling layer as before. The output was fed into a classifier with two fully connected layers, each having 4,096 units and each followed by a DropOut layer (*p* = 0.5). We used a 128 × 128 input size, and the *ReLU* activation function in all layers.

The age regression network was comprised of two 3 × 3 convolutional layers, each followed by a max pooling layer, and a single fully connected layer with 2,048 units. We retained the 128 × 128 input size and the *ReLU* activation function for this task.

Performing deep learning with small datasets can be particularly challenging, as small training sets can be easy for a deep network to memorize, resulting in problematic overfitting. This often results in low training error, but high testing error. This discrepancy is termed the *generalization error*. One way to protect against overfitting when performing learning with images is to perform dataset augmentation. By applying distortions to images in the training set with some probability, the size of the training set is artificially but effectively increased. In all experiments, brightness, contrast, cropping, and flipping distortions were applied randomly to augment the training set.

For testing, a random 80–20 train-test split was used in all experiments. It is considered good practice to implement “early stopping” during training, by withholding a portion of the dataset (called the *validation set*) to test on and stopping training once the network attains a certain level of performance on these samples. This helps to prevent overfitting, where performance on the test set may subsequently drop as training continues past this point. Since the 80–20 split used by previous published results does not include any validation set that could be used to implement early stopping, we stop training after the training loss appears to plateau. The gradient-adaptive Adam algorithm was used for optimization in all experiments (Kingma and Ba, 2015).

## 4. Results

**Figure 4:**
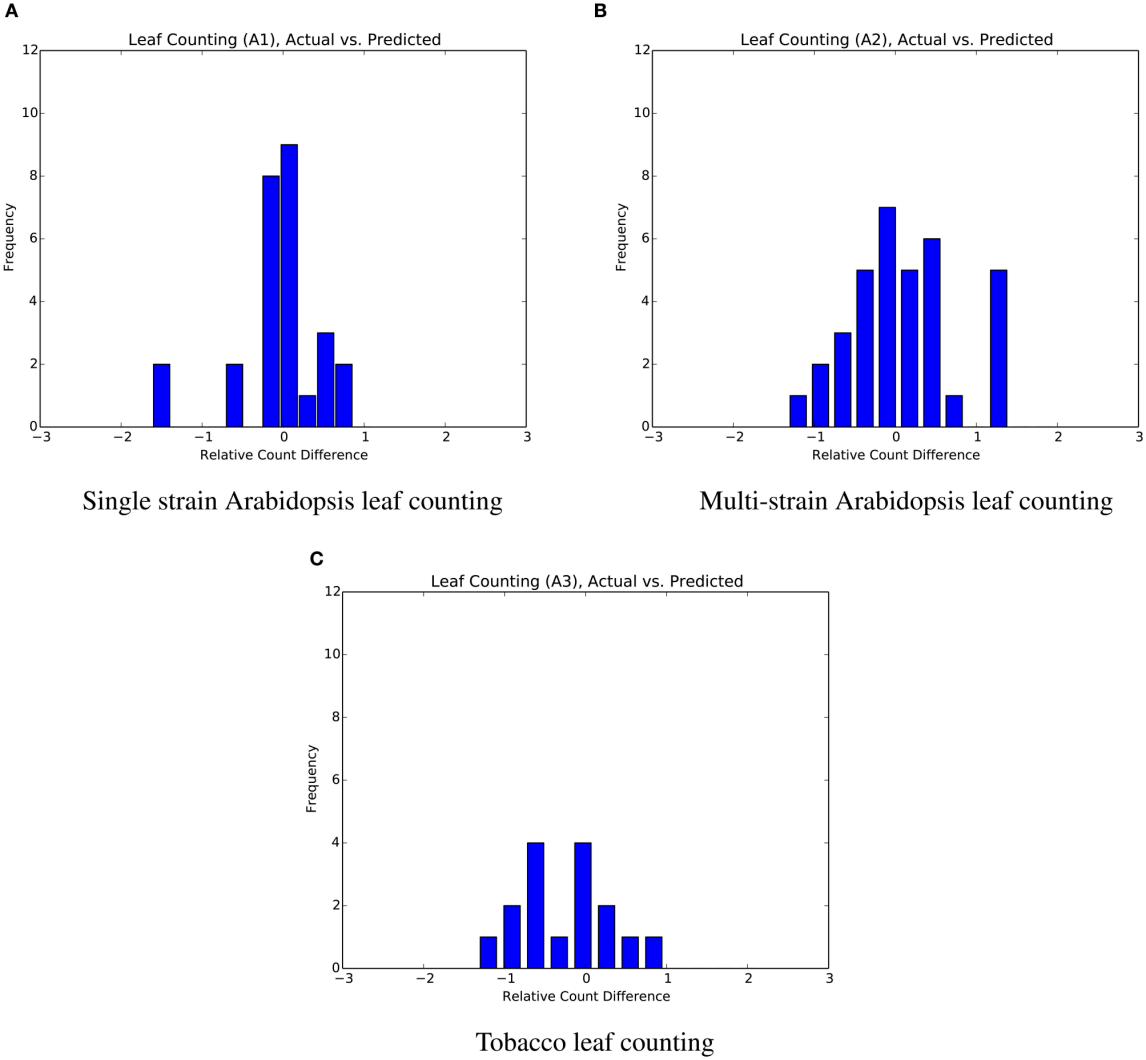
Distribution of count errors for the three leaf counting datasets. The output of the regressor is not rounded in order to produce more granular bins. **(A)** Single strain Arabidopsis leaf counting. **(B)** Multi-strain Arabidopsis leaf counting. **(C)** Tobacco leaf counting.

**Figure 5:**
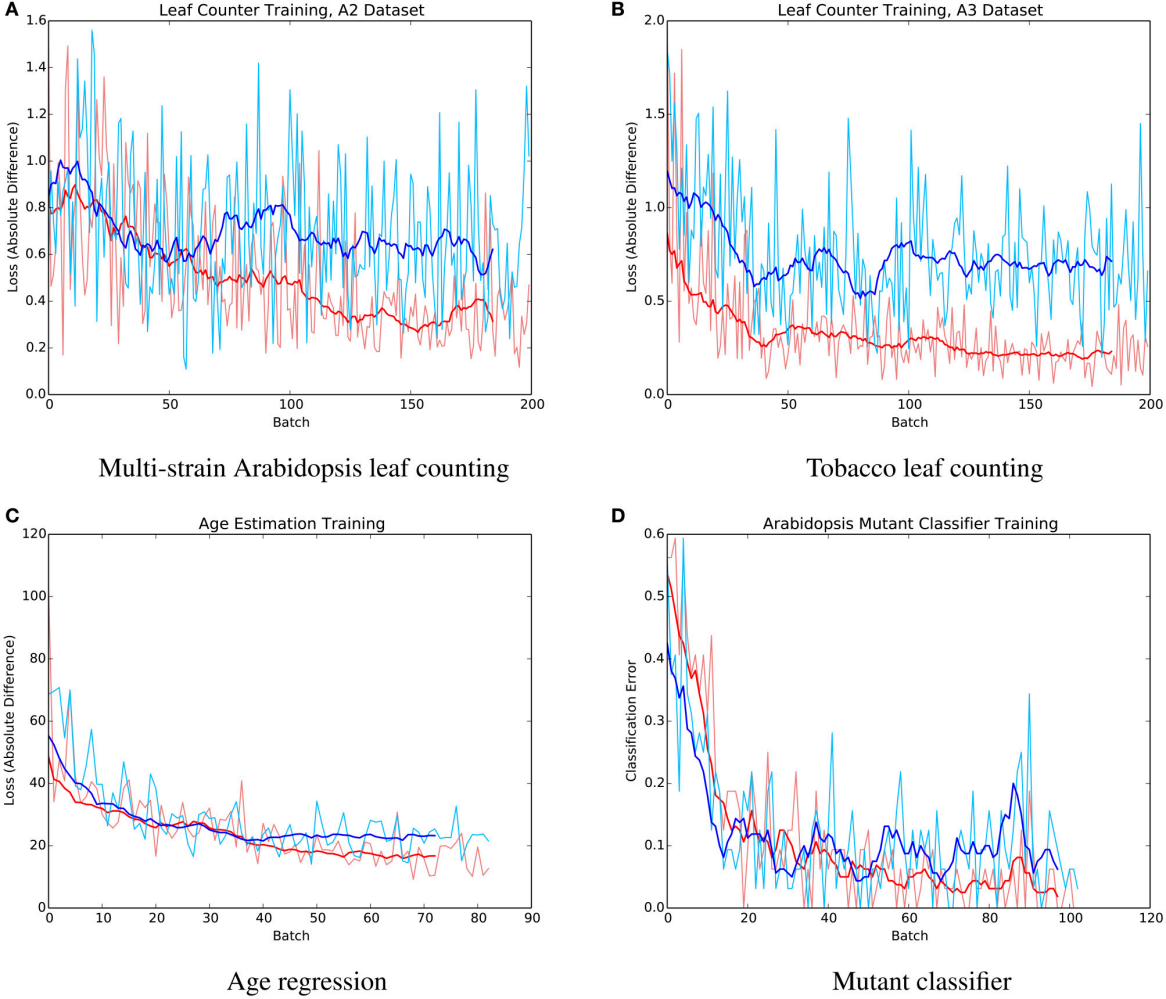
Example training (red) and testing (blue) curves for several benchmark tasks. **(A)** Multi-strain Arabidopsis leaf counting. **(B)** Tobacco leaf counting. **(C)** Age regression. **(D)** Mutant classifier.

The mean absolute difference results for the three different leaf counting datasets are provided in Table 2 and detailed histograms of errors are shown in Figure 4. We compared the performance against a result from the literature using an unsupervised machine learning method (Giuffrida et al., 2015), and reproduced their comparison against a counting-by-segmentation method from the literature (Pape and Klukas, 2015). Unlike the authors of the cited studies, we do not include results for training accuracy, because a deep convolutional network with sufficient capacity is able to fit the training data with an arbitrary degree of accuracy. We also do not report the (non-absolute) count difference (*CountDiff*), which does not directly measure performance since over-prediction and under-prediction are able to negate each other. Training and testing curves for Arabidopsis leaf counting, age regression, and mutant classification are shown in Figure 5.

**Table 2:** Mean (std) absolute difference for the three leaf counting benchmarks.

	**Giuffrida et al., 2015**	**Pape and Klukas, 2015**	**Proposed**
A1	1.27 (1.15)	2.2 (1.3)	0.41 (0.44)
A2	2.44 (2.28)	1.2 (1.3)	0.61 (0.47)
A3	1.36 (1.37)	2.8 (2.5)	0.61 (0.54)

The mutant classifier model proved effective in distinguishing between five different mutants of *Arabidopsis*, with a measured 96.88% mean test accuracy. This is an encouraging result, and it sets the baseline performance for this task as the first published result.

For the age regression task, our model achieves a mean absolute difference of 20.8 h with a standard deviation of 14.4 h. The ground truth labels for this task range between 392 and 620 h. Like for the mutant classification task, this result is the first published result for this task.

The results for the experiment investigating robustness to variance in scene were a mean absolute difference of 0.64, with a standard deviation of 0.51. These results are comparable with the unmodified test set (Table 2) which suggests that the network is indeed robust to changes in lighting conditions.

## 5. Discussion

For the leaf counting task, the proposed method shows significantly better performance on each of the three benchmark datasets in terms of the absolute difference in count compared to previous methods. In addition, both the mean and standard deviation are more consistent between tasks using the proposed method. Both results from the literature show significantly degraded performance on a selection the three benchmark tasks—multi-accession Arabidopsis for Giuffrida et al. (2015), and both Col-0 Arabidopsis and tobacco for Pape and Klukas (2015). In contrast, the proposed CNN method shows that it is capable of learning representations of the training data which are effective for each of the three datasets.

Tests with artificially modulated image brightness and contrast demonstrate that the CNN method can be made robust to changes in scene lighting conditions through data augmentation during training. Therefore, the proposed technique has better potential than classical image analysis methods for translation to field phenotyping where scene conditions are more variable, e.g., for measuring emergence counts from aerial images of rosettes in field plots. It also means that the method can be used in indoor environments such as greenhouses, where the lighting modality cannot be controlled.

**Figure 6:**
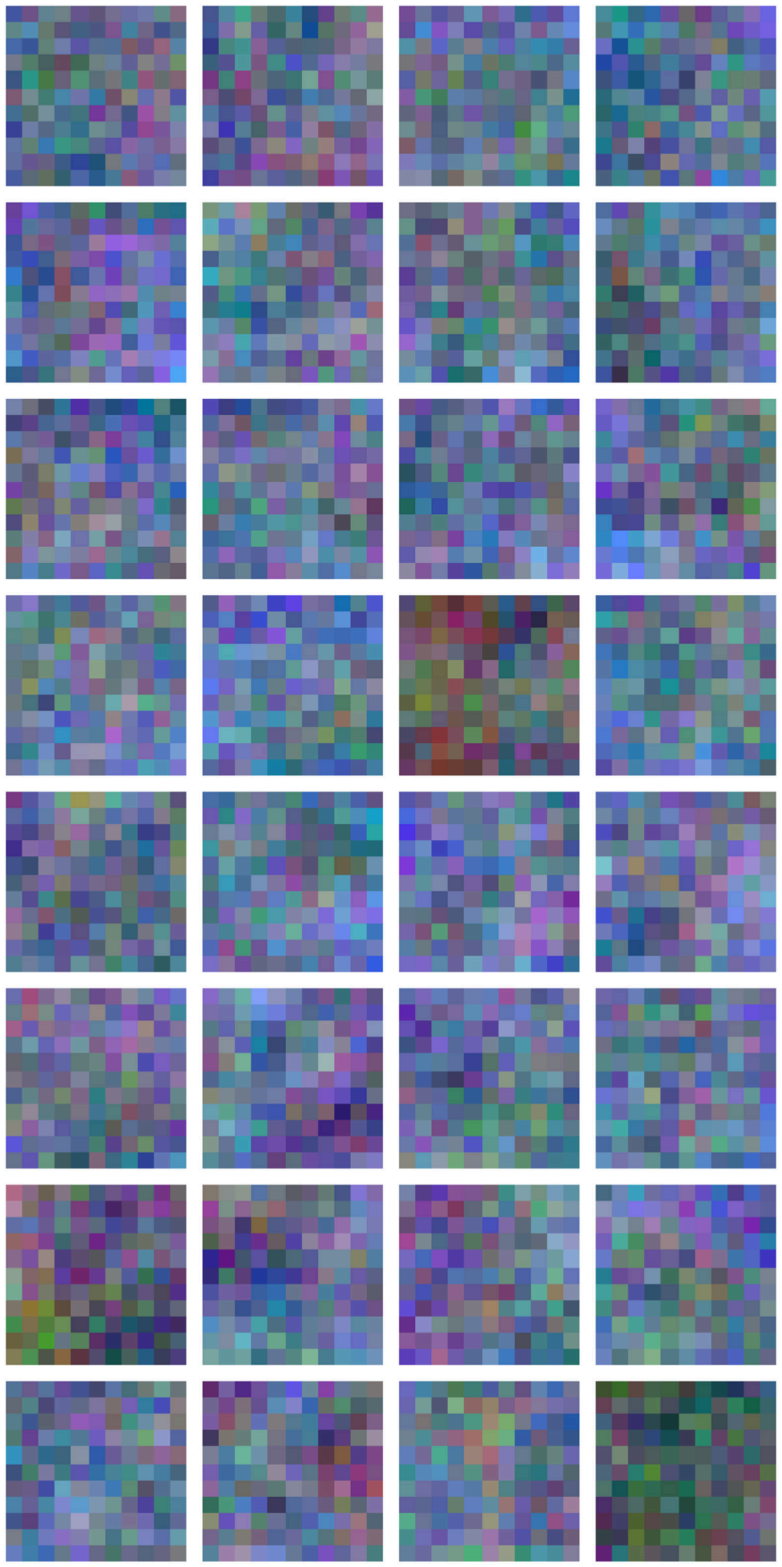
Visualization of filter weights in the first convolutional layer of a leaf counting network.

It is common to visualize the filters of the first convolutional layer, since these filters often contain some interpretable structure as they correspond to operations over the input image. Later convolutional layers are more difficult to interpret, as they correspond to abstract output from the previous convolutional layer. Since the leaf counter network uses 5 × 5 × 3 filter weights, not much interesting structure appears in the filter weights of the first convolutional layer during training. However, by increasing the filter size to 11 × 11 × 3, some interesting structure appears in these filters (Figure 6). The trained filters result in mostly green and violet pixels. Violet pixels respond to high values in the red channel and low values in the green channel; therefore, it is likely that the presence of leaves is being suppressed in these regions of the receptive field.

It is noteworthy that both previous leaf counting algorithms to which we compare our method (Table 2) require pre-segmented plant images, and presumably the performance of their algorithm is dependent on the accuracy of this segmentation. In contrast, the CNN technique requires no such pre-processing and only the raw RGB images are required as input. The authors of the dataset describe the inputs to the age estimation task to be the RGB images as well as the labels for mutant type; however, we use only the RGB images and rely on the network to learn representations which are robust to differences in growth rate between mutants. Experiments using the mutant labels actually performed worse, as it allowed the network to use the label to fit the training data more aggressively and this was detrimental to generalization performance.

The advantage of supervised learning methods over hand-engineered image analysis techniques in tasks such as leaf counting is their capacity for complex representation learning. For example, a hand-engineered image processing pipeline must be designed to accommodate leaves of different shapes and sizes (for plants of different ages and genotypes), leaves with different length petioles, as well as partially overlapping leaves. A supervised representation learning algorithm such as a CNN is capable of automatically learning a representation of the data which takes into account all of these factors, and any others which are present in the training data.

Although designing CNN architectures requires less hand-engineering than image processing pipelines, the process is not completely automated. Building a network architecture to perform any computer vision task involves some iterative optimization in two areas: the number and size of network layers, and the values of hyperparameters such as learning rate and regularization strength. For hyperparameter tuning, some automated methods are available such as simple grid search and Bayesian optimization (Snoek et al., 2012). Although training a CNN requires such considerations, it is less cumbersome than tuning the alternative image processing pipeline. For example, the leaf counting pipeline described in Pape and Klukas (2015) contains 14 discrete image processing steps, the majority of them having tuneable parameters such as *noise area limit* and *gap fill size limits*.

There are several promising directions for future research for which DPP software development is ongoing. Support for additional types of network architectures, such as Residual Networks (He et al., 2016), may offer more utility for future applications. Detection and localization of plants and leaves, a natural progression to the leaf counting regression discussed in this paper, could be made possible with architectures such as Fast-RCNN (Girshick, 2015). In addition, implementing recurrent models such as Long Short Term Memory (LSTM) networks (Hochreiter and Schmidhuber, 1997) would allow for the prediction of temporal features such as growth rate, which are an important class of features. Implementing transfer learning in the platform has the potential to provide higher accuracy and lower training times. Transfer learning involves starting with a network pre-trained on large datasets, such as the ImageNet database (Deng et al., 2009), and then fine-tuning the network with a smaller set of images tailored to the task of interest, e.g., rosette images. This technique is widely accepted in the literature for bootstrapping the learning process, and has proven successful in plant disease diagnosis (Mohanty et al., 2016).

Although the DPP platform has only been tested with data collected in a controlled environment, further testing can be done to explore applications with outdoor, field-level applications. There are also opportunities to test the performance of the system on larger datasets, such as those collected from automated greenhouses. Finally, we look forward to the applications, collaborations, and suggestions put forward by the plant phenotyping community as the platform matures.

## 6. Summary

In this work, we introduced a deep learning platform for image-based plant phenotyping called Deep Plant Phenomics. We demonstrated its effectiveness by performing three image-based phenotyping tasks from the literature. Our approach achieved state of the art performance on the leaf counting task and set baseline results for the mutant classification and age regression tasks. The software has been released as an open-source package, with the goal of promoting the use of deep learning within the plant phenotyping community.

## Author contributions

The software and experiments were implemented by JU. The manuscript was written jointly by JU and IS.

### Conflict of interest statement

The authors declare that the research was conducted in the absence of any commercial or financial relationships that could be construed as a potential conflict of interest.
