# Corrigendum: Deep Plant Phenomics: A Deep Learning Platform for Complex Plant Phenotyping Tasks

**DOI:** 10.3389/fpls.2017.02245

**Published:** 2018-01-15

**Authors:** Jordan R. Ubbens, Ian Stavness

**Affiliations:** Department of Computer Science, University of Saskatchewan, Saskatoon, SK, Canada

**Keywords:** phenotyping, deep learning, methods, computer vision, machine learning

The dataset referred to as the *IPPN dataset* (p. 4) in the original article is now referred to by its authors as the *PRL dataset*^1^. This dataset was used because it includes annotations for all three of the tasks performed in the validation experiments^2^. In the results on the leaf counting task (Table 2), the proposed method was compared against two results from the literature. However, the results reported in the cited papers were performed on a different version of the dataset, which is referred to by its authors as *CVPPP 2015_LCC* (for the leaf counting competition of the 2015 Computer Vision Problems in Plant Phenotyping workshop). Therefore, the direct comparison is not warranted. However, *R*^2^ between the actual and predicted leaf counts for the Plant/Ara2012, Plant/Ara2013-Canon, and Plant/Tobacco leaf counting datasets as presented in the article are 0.85, 0.90, and 0.74, respectively, demonstrating strong performance without the context of this comparison. This error does not change the scientific conclusions of the article in any way.

## Conflict of interest statement

The authors declare that the research was conducted in the absence of any commercial or financial relationships that could be construed as a potential conflict of interest.

## Footnotes

^1^https://www.plant-phenotyping.org/datasets-home.

^2^Datasets A1, A2, and A3 in the original article refer to datasets Plant/Ara2012, Plant/Ara2013-Canon, and Plant/Tobacco, respectively in the PRL data.

